# Ethnicity modifies the relation between fasting plasma glucose and HbA_1c_ in Indians, Malays and Chinese

**DOI:** 10.1111/j.1464-5491.2012.03599.x

**Published:** 2012-07

**Authors:** K Venkataraman, S L Kao, A C Thai, A Salim, J J M Lee, D Heng, E S Tai, E Y H Khoo

**Affiliations:** 1 Department of Obstetrics and GynaecologySingapore; 2 Department of MedicineSingapore; 3 Department of Epidemiology and Public Health, Yong Loo Lin School of Medicine, National University of SingaporeSingapore; 4 Ministry of Health, Epidemiology and Disease Control DivisionSingapore

**Keywords:** diabetes mellitus, diagnosis, ethnicity, glycated haemoglobin

## Abstract

**Aims:**

To study whether HbA_1c_, and its relationship with fasting plasma glucose, was significantly different among Chinese, Malays and Indians in Singapore.

**Methods:**

A sample of 3895 individuals without known diabetes underwent detailed interview and health examination, including anthropometric and biochemical evaluation, between 2004 and 2007. Pearson’s correlation, analysis of variance and multiple linear regression analyses were used to examine the influence of ethnicity on HbA_1c_.

**Results:**

As fasting plasma glucose increased, HbA_1c_ increased more in Malays and Indians compared with Chinese after adjustment for age, gender, waist circumference, serum cholesterol, serum triglyceride and homeostasis model assessment of insulin resistance (*P*-interaction < 0.001). This translates to an HbA_1c_ difference of 1.1 mmol/mol (0.1%, Indians vs. Chinese), and 0.9 mmol/mol (0.08%, Malays vs. Chinese) at fasting plasma glucose 5.6 mmol/l (the American Diabetes Association criterion for impaired fasting glycaemia); and 2.1 mmol/mol (0.19%, Indians vs. Chinese) and 2.6 mmol/mol (0.24%, Malays vs. Chinese) at fasting plasma glucose 7.0 mmol/l, the diagnostic criterion for diabetes mellitus.

**Conclusions:**

Using HbA_1c_ in place of fasting plasma glucose will reclassify different proportions of the population in different ethnic groups. This may have implications in interpretation of HbA_1c_ results across ethnic groups and the use of HbA_1c_ for diagnosing diabetes mellitus.

## Introduction

Glycated haemoglobin (HbA_1c_), the fraction of haemoglobin non-enzymatically linked to glucose (glycated) at the valine terminal of the β-chain, is widely used as an indicator of glycaemic status in the management of diabetes mellitus. The level of HbA_1c_ is proportional to the blood glucose levels and reflects the glycaemic status over the preceding 3 months (the average lifespan of erythrocytes) [1,2]. Data from large randomized controlled trials comparing intensive and conventional glycaemic control have been used to define therapeutic targets for HbA_1c_ that guide diabetes management [3–6].

An International Expert Committee, the American Diabetes Association and the World Health Organization have now recommended the use of HbA_1c_ as a diagnostic tool for diabetes mellitus [7–9]. The measurement of HbA_1c_ was favoured as it exhibited less intra-individual variability, greater analytical stability and also obviated the need for fasting. In addition, HbA_1c_ assays are also being standardized worldwide. It has also been demonstrated that HbA_1c_ performs comparably with fasting and 2-h postprandial glucose measures in identifying individuals with and without retinopathy [7–11]. The criteria recommended by the American Diabetes Association are HbA_1c_ ≥ 39 mmol/mol (5.7%) for increased risk for diabetes and ≥ 48 mmol/mol (6.5%) for diabetes.

Several studies have demonstrated that individuals may exhibit different HbA_1c_ at the same glucose level [12–14]. In particular, some ethnic groups appear to have higher HbA_1c_ levels compared with others at the same levels of plasma glucose [14–17]. The use of HbA_1c_ to diagnose diabetes has been shown to under- or overestimate the prevalence of diabetes in comparison with glucose-based criteria in different populations or ethnic groups [17–20]. This indicates that the relationship between HbA_1c_ and blood glucose may not be similar across ethnic groups and populations.

Delineating ethnic differences in the relationship between plasma glucose and HbA_1c_ is especially important in Asian populations, given that the prevalence of cases of diabetes in this region is projected to more than double by 2030 [21,22].

In this study, we examined the relationship between fasting plasma glucose and HbA_1c_ among the three major ethnic groups, Chinese, Malay and Indians living in Singapore, whose prevalence rates for diabetes mellitus are relatively high at 7.1, 11 and 15.3%, respectively [23].

## Patients and methods

### Study population

Individuals who had participated previously in one of four population-based cross-sectional studies were invited to be part of this study (Singapore Prospective Study). The four studies include: the Thyroid and Heart Study 1982–1984 [24], the National Health Survey 1992 [25], the National University of Singapore Heart Study 1993–1995 [26] and the National Health Survey 1998 [27] and have been described previously [28]. Briefly, all studies were a random sample of individuals from the Singapore population, with disproportionate sampling stratified by ethnicity to increase the number of the minority ethnic groups (Malays and Asian Indians). Subjects deceased at time of follow-up (as shown by data-linkage to Registry of Births and Deaths) were excluded (*n* = 517). Also excluded were six subjects who had emigrated and 85 subjects who had errors in the records for the identity card number.

Subjects were contacted to obtain an appointment for investigators to administer the questionnaire at the subject’s home. Three home visits were made on three different occasions, including one weekend day and one weekday, before a subject was deemed non-contactable. In total, 2673 subjects were non-contactable and, of the remaining subjects, 30 (0.3%) refused to participate. All subjects were invited to attend a health examination for additional tests and collection of biological specimens.

A total of 10 633 individuals were invited; 7742 subjects (response rate 74.1%) completed the health questionnaire, of which 5157 (66.6% of those who completed the questionnaire or 49.4% of all eligible subjects) also attended the health examination. Ethics approval was obtained from two Institutional Review Boards (National University of Singapore and Singapore General Hospital). Informed consent was obtained before commencement of the study.

### Data collection

Data on demographic and lifestyle (alcohol intake, smoking) factors, as well as medical history (including physician-diagnosed hypertension, diabetes mellitus and hyperlipidaemia) were collected using interviewer-administered questionnaires. For the health examination, participants were examined in the morning following a 10-h overnight fast. Venous blood was drawn and collected in plain and fluoride oxalate tubes and stored at 4 °C for a maximum of 4 h prior to processing.

All biochemical analyses on blood were carried out at the National University Hospital Referral Laboratory, which is accredited by the College of American Pathologists. Serum total cholesterol, triglyceride and HDL cholesterol levels were measured using an automated autoanalyser (ADVIA 2400; Bayer Diagnostics, Tarrytown, NY, USA). LDL cholesterol levels were calculated using the Friedewald formula.

Plasma glucose was also assayed using enzymatic methods (ADVIA 2400; Bayer Diagnostics) using blood collected in fluoride oxalate tubes. Plasma creatinine was measured by enzymatic methods (reaction of Tanganelli) and implemented on an ADVIA 2400 chemistry system. The intra- and interday variability for total cholesterol, triglycerides, HDL cholesterol, plasma glucose and creatinine was 0.80–1.57, 0.93–1.15, 0.00–3.85, 1.27–3.4, 0.56–0.65, 1.18–2.00, 0.00–0.93, 1.68–1.83, 2.50–6.60 and 5.60–7.20%, respectively.

HbA_1c_ was measured using high-pressure liquid chromatography on a Biorad Variant II analyser (Bio-Rad Laboratories, Hercules, CA, USA), an assay that was accredited by the National Glycoprotein Standardization Program with controls traceable to the Diabetes Control and Complications Trial. The intra- and interday coefficients of variability for HbA_1c_ were 0.0–2.0 and 0.85–1.54%, respectively.

Height was measured without shoes using a wall-mounted stadiometer. Weight was measured in light clothing using the same digital scale (SECA, model 782 2321009; Vogel & Halke, Hamberg, Germany). Participants were instructed to remove any objects such as keys and mobile phone before measurement. Two readings of blood pressure were taken from participants after 5 min of resting using an automated blood pressure monitor (Dinamap Pro100V2; Criticon, Norderstedt, Germany). A third reading was performed if the difference between two readings of systolic blood pressure was greater than 10 mmHg or of diastolic blood pressure was greater than 5 mmHg. Mean values of the closest two readings were calculated. The inter- and intra-observer coefficient of variation for systolic blood pressure was 0.51–10.2 and 0-2.5%, whilst it was 0.41–7.5 and 0–2.5% for diastolic blood pressure. Waist circumference was measured midway between the lower rib margin and the iliac crest and hip circumference was measured at the widest point over the greater trochanters. The waist–hip ratio was calculated by dividing waist circumference (in cm) by hip circumference (in cm).

Homeostasis model assessment of insulin resistance (HOMA-IR) was calculated as [fasting insulin (μU/ml) × fasting glucose (mmol/l)]/22.5.

Known diabetes was defined as history of diabetes and/or currently taking anti-diabetic agents.

## Statistical analysis

Of the 5157 individuals who completed both the study questionnaire and the health examination, those with known diabetes mellitus (*n* = 325), without measured HbA_1c_ values (*n* = 932) and without recorded ethnicity (*n* = 2) were excluded. The analysis reported in this paper is based on data from 3895 subjects without previous diagnosis of diabetes and with measured HbA_1c_ values. Descriptive analysis was used to study the baseline characteristics. Variables with non-linear distributions were log-transformed to ensure linearity, as needed. Analysis of covariance was used to obtain adjusted means. The step-down Bonferroni method was used to adjust for multiple comparisons. Pearson’s correlation and one-way analysis of variance (anova) were used to identify continuous and categorical variables significantly influencing HbA_1c_. Multiple linear regression was used to further clarify the effect of these variables on HbA_1c_. Fasting plasma glucose was centred to the mean for regression analysis. Two dummy variables were created and used to study the interaction between ethnicity and fasting plasma glucose in relation to HbA_1c_, by incorporating the cross-product term in the model.

All unweighted statistical analysis was conducted using Predictive Analytics SoftWare (PASW) Statistics (version 18; IBM, Chicago, IL, USA). Weighted analysis to test the robustness of interactions was conducted using R (version 2; R Foundation for Statistical Computing, Vienna, Austria). All statistical tests used were two-sided, with *P* < 0.05 being considered as significant. All values are mean (standard error) unless otherwise specified.

## Results

Baseline characteristics of the population studied are shown in Table 1. The mean age was 49 (12) years, with almost equal gender distribution. The majority were of Chinese ethnicity (2697, 69.2%), followed by Malays (633, 16.3%) and Indians (565, 14.5%). The mean HbA_1c_ was 40 ± 9.1 mmol/mol (5.8% ± 0.83), with mean fasting plasma glucose and insulin values of 4.94 mmol/l (± 1.16) and 7.85 mU/l (± 6.31).

**Table 1:** Participant characteristics by ethnic group in the Singapore Prospective Study Program, 2004–2007

Variable	All	Chinese (*n* = 2697, 69.2%)	Malay (*n* = 633, 16.3%)	Indian (*n* = 565, 14.5%)	*P*
Age	49 (0.2)	49 (0.2)	49 (0.5)	51 (0.5)	0.005
Male	1853 (47.6)	1263 (46.8)	312 (49.4)	276 (48.8)	0.411
BMI (kg/m^2^)‡	23.7 (0.1)	22.8 (0.1)	25.8 (0.2)***	25.8 (0.2)***	< 0.001
Waist–hip ratio‡	0.85 (0.001)	0.84 (0.001)	0.85 (0.003)*	0.87 (0.003)***†††	< 0.001
Waist circumference (cm)‡	83.6 (0.2)	81.5 (0.2)	86.8 (0.4)***	89.9 (0.4)***†††	< 0.001
Systolic blood pressure (mmHg)‡^a^	131 (0.3)	130 (0.3)	136 (0.7)***	131 (0.7)†††	< 0.001
Diastolic blood pressure (mmHg)‡	78 (0.2)	77 (0.2)	79 (0.4)***	79 (0.4)**	< 0.001
HbA_1c_ (mmol/mol)	40 (0.1)	40 (0.2)	42 (0.3)	43 (0.3)	< 0.001
HbA_1c_ (%)‡	5.84 (0.01)	5.77 (0.02)	5.95 (0.03)***	6.06 (0.03)***†	
Fasting plasma glucose (mmol/l)‡	4.94 (0.02)	4.84 (0.02)	5.10 (0.04)***	5.24 (0.05)***	< 0.001
Insulin (mU/l)‡	7.85 (0.1)	7.03 (0.12)	8.24 (0.24)***	11.32 (0.26)***†††	< 0.001
HOMA-IR‡	1.79 (0.03)	1.56 (0.03)	1.90 (0.07)***	2.72 (0.07)***†††	< 0.001
Cholesterol (mmol/l)‡	5.27 (0.02)	5.22 (0.02)	5.53 (0.04)***	5.24 (0.04)†††	< 0.001
Triglycerides (mmol/l)‡	1.35 (0.01)	1.31 (0.02)	1.47 (0.03)***	1.41 (0.03)*	< 0.001
HDL cholesterol (mmol/l)‡	1.44 (0.01)	1.49 (0.01)	1.39 (0.01)***	1.25 (0.01)***†††	< 0.001
LDL cholesterol (mmol/l)‡	3.22 (0.01)	3.14 (0.02)	3.47 (0.03)***	3.35 (0.03)***†	< 0.001
Creatinine (μmol/l)‡	79.97 (0.31)	78.99 (0.29)	81.77 (0.59)***	82.65 (0.62)***	< 0.001

HOMA-IR, homeostasis model assessment of insulin resistance.

* *P* < 0.05,

** *P* < 0.01,

*** *P* < 0.001 (Chinese as reference);

† *P* < 0.05,

††*P* < 0.01,

††† *P* < 0.001 (Malay as reference) by anova using step-down Bonferroni correction for multiple comparisons.

‡ Means adjusted for age and gender.

All values are means (± se) unless otherwise specified.

**FIGURE 1:**
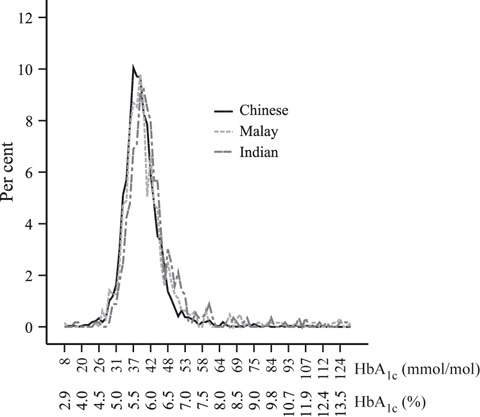
Distribution of HbA_1c_ by ethnic group in the Singapore Prospective Study Program, 2004–2007

There were significant differences between the three ethnic groups in the variables measured. Both Malays and Indians had higher mean BMI and serum creatinine than the Chinese (*P* < 0.001), adjusted for age and gender. Indians had higher age- and gender-adjusted mean waist circumference and waist–hip ratio compared with the other ethnic groups (*P* < 0.001). Malays had higher mean systolic blood pressure and serum cholesterol compared with Chinese and Indians (*P* < 0.001). Indians had the highest mean fasting insulin levels (*P* < 0.001), followed by the Malay (*P* < 0.001), compared with the Chinese. There were significant differences (*P* < 0.05) in unadjusted HbA_1c_ between the three ethnic groups, with Indians having the highest mean HbA_1c_ (43 mmol/mol, 95% CI 42–44; 6.09%, 95% CI 5.99–6.18), followed by Malays (42 mmol/mol, 95% CI 41–43; 5.96%, 95% CI 5.87–6.05) and Chinese (39 mmol/mol, 95% CI 39–40; 5.76%, 95% CI 5.74–5.79). The distribution of HbA_1c_ was shifted towards the right in Indians and Malays, compared with the Chinese across the range of HbA_1c_ values, suggesting that these differences were not just attributable to a higher proportion of undiscovered diabetes mellitus in the Indians and Malays (Fig. 1).

**FIGURE 2:**
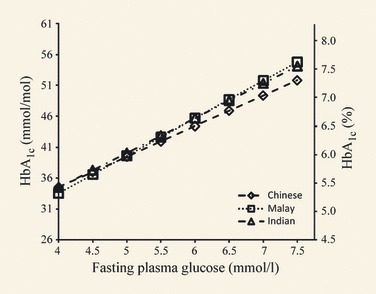
Estimated mean HbA_1c_ at different fasting plasma glucose values, the Singapore Prospective Study Program, 2004–2007. Mean HbA_1c_ estimated after adjusting for age, gender, ethnicity, fasting plasma glucose–ethnicity interaction, waist circumference, serum cholesterol, triglyceride and homeostasis model assessment of insulin resistance (HOMA-IR) at each fasting plasma glucose level.

On univariate analysis using Pearson’s correlation, fasting plasma glucose (*r* = 0.79, *P* < 0.001), age (*r* = 0.208, *P* < 0.001), BMI (log-transformed; *r* = 0.21, *P* < 0.001), waist circumference (*r* = 0.23, *P* < 0.001), waist–hip ratio (*r* = 0.18, *P* < 0.001), systolic blood pressure (*r* = 0.19, *P* < 0.001), diastolic blood pressure (*r* = 0.15, *P* < 0.001), total cholesterol (*r* = 0.13, *P* < 0.001), serum triglyceride (log-transformed; *r* = 0.20, *P* < 0.001), fasting insulin (*r* = 0.17, *P* < 0.001) and HOMA-IR (log-transformed; *r* = 0.34, *P* < 0.001) were significantly associated with HbA_1c_. Age, gender, ethnicity, waist circumference, fasting plasma glucose, total cholesterol, triglycerides and HOMA-IR continued to be significantly associated with HbA_1c_ on stepwise multiple linear regression, although the magnitudes of the main effects were small for all variables except fasting plasma glucose (Table 2). Fasting plasma glucose showed the strongest association with HbA_1c_, explaining 62.4% of the variance for HbA_1c_. In addition, a statistically significant interaction between fasting plasma glucose and ethnicity was noted (*P*-interactions < 0.001). As fasting plasma glucose increased, HbA_1c_ increased more in Malays and Indians compared with Chinese. Subsequently, we conducted robust analysis to reduce the effect of outliers, which gave similar results for the interaction between ethnicity and fasting plasma glucose (data not shown). Estimation of the mean HbA_1c_ values over a range of fasting plasma glucose (4–7.5 mmol/l) showed that Malays and Indians appear to have lower HbA_1c_ at low fasting plasma glucose values and higher HbA_1c_ at higher values of fasting plasma glucose, compared with the Chinese, with crossover at fasting plasma glucose 5.0 mmol/l (Fig. 2). This translates to a difference of 1.1 mmol/mol (95% CI 0.5–1.6; 0.1%, 95% CI 0.05–0.15; Indians vs. Chinese) and 0.9 mmol/mol (95% CI 0.3–1.4; 0.08%, 95% CI 0.03–0.13; Malays vs. Chinese) at fasting plasma glucose 5.6 mmol/l, the American Diabetes Association criterion for impaired fasting glycaemia; and a difference of 2.1 mmol/mol (95% CI 1.2–3.0; 0.19%, 95% CI 0.11–0.27; Indians vs. Chinese) and 2.6 mmol/mol (95% CI 1.7–3.5; 0.24%, 95% CI 0.16–0.32; Malays vs. Chinese) at fasting plasma glucose 7.0 mmol/l, the diagnostic criterion for diabetes mellitus.

**Table 2:** Multivariate regression model with HbA_1c_ as dependent variable; the Singapore Prospective Study Program, 2004–2007

				95% CI	
				
Variables in model	β-coefficient	Standard error	*P*	Lower	Upper
Intercept	5.078	0.092	< 0.001	4.942	5.324
Age	0.005	0.001	< 0.001	0.003	0.006
Female	0.085	0.018	< 0.001	0.049	0.12
Indian	0.055	0.025	0.024	0.007	0.103
Malay	0.005	0.023	0.813	−0.039	0.05
Fasting plasma glucose (centred)	0.493	0.012	< 0.001	0.469	0.517
Waist circumference	0.004	0.001	< 0.001	0.002	0.005
Serum triglyceride (log)	0.14	0.043	0.001	0.056	0.224
Serum cholesterol	0.033	0.009	< 0.001	0.015	0.052
HOMA-IR (log)	-0.072	0.036	0.043	−0.142	−0.002
Indian × fasting plasma glucose (centred)	0.066	0.017	< 0.001	0.033	0.1
Malay × fasting plasma glucose (centred)	0.133	0.017	< 0.001	0.079	0.147

*R*^2^ = 0.632.

HOMA–IR, homeostasis model assessment of insulin resistance.

## Discussion

In this study, we have shown that blood glucose is the major determinant of HbA_1c_ in Chinese, Malays and Indians living in Singapore. This is in line with previous studies [1,29,30]. The novelty of our study is the demonstration that the relationship between fasting plasma glucose and HbA_1c_ differs between the three major ethnic groups living in Singapore. This is particularly relevant as these ethnic groups are closely related to the majority of the population living in Asia, where we anticipate a large increase in the prevalence of diabetes in the next several decades. HbA_1c_ values were lower in Malays and Indians compared with Chinese at lower fasting plasma glucose levels, with crossover at fasting plasma glucose of around 5 mmol/l. At fasting plasma glucose levels above 5 mmol/l, HbA_1c_ values were higher in Malays and Indians compared with Chinese, with greater differences at higher fasting plasma glucose concentrations. To illustrate the relevance of these findings to clinical practice, we estimated that ethnicity contributed to a difference in HbA_1c_ of 2.1–2.6 mmol/mol (0.19–0.24%) at the fasting plasma glucose cut-off of 7.0 mmol/l, the current level for the diagnosis of diabetes mellitus. If a single HbA_1c_ cut-off is used to diagnose diabetes (and pre-diabetes) across these three ethnic groups, it will reclassify more Indians and Malays as having these conditions than Chinese, compared with fasting plasma glucose-based criteria. Although the effect of ethnicity in HbA_1c_ seems small, it may be significant if HbA_1c_ is widely accepted and applied as a diagnostic tool in our population. To provide some context, it is useful to note that 4% of the population has an HbA_1c_ between 48 and 50 mmol/mol (6.5–6.7%).

Several previous studies have described ethnic differences in HbA_1c_, but only a few have examined for an interaction between ethnicity and glucose in determining HbA_1c_. Herman *et al*. showed differences in HbA_1c_ in subjects with impaired glucose tolerance of white, Hispanic, Asian, Indian American and African American ethnicities. An HbA_1c_ difference of 0.15–0.4%, a magnitude that is similar to that found in our study, persisted across ethnic groups after adjusting for age, gender, systolic and diastolic blood pressures, BMI, glucose and insulin levels [14]. In another study, Ziemer *et al*. demonstrated that African Americans had higher HbA_1c_ levels compared with white Caucasians across a range of glucose levels. Similar to our study, they found that these differences increased with an increase in glucose levels [15]. However, neither of these studies formally tested for an interaction between ethnicity and glucose in determining HbA_1c_. Jorgensen *et al*. reported that Greenland Inuit had significantly higher levels of HbA_1c_ than Danes at any level of glycaemia. However, they did not find that ethnicity modified the association between plasma glucose and HbA_1c_ in their analysis [17]. In another study of white and African American subjects with diabetes, Bleyer *et al*. reported an interaction between ethnicity and random serum glucose for HbA_1c_. However, this study was limited to individuals with established diabetes [16]. Although we excluded individuals with known diabetes in our study, fasting plasma glucose values ranged from 2.7 to 21.5 mmol/l. Thus, we were able to demonstrate an interaction between ethnicity and glucose levels in relation to HbA_1c_ across a larger range of glycaemia than has been studied previously.

We have a few hypotheses that may explain this differential relationship between HbA_1c_ and fasting plasma glucose across ethnic groups. Firstly, there could be differences in the daily glycaemic exposure (such as postprandial glucose excursions) amongst the three groups at the same fasting plasma glucose. This reflects the failure of fasting plasma glucose to accurately represent an individual’s glycaemic exposure, a finding confirmed by the A1c-Derived Average Glucose (ADAG) Study [30].

Secondly, factors independent of glycaemia may influence HbA_1c_ levels to different extents in separate ethnic groups. Although HbA_1c_ and blood glucose levels are highly correlated, glucose exposure only explains a portion of the variability in HbA_1c_. In the A1c-Derived Average Glucose study, the average estimated glucose obtained from continuous glucose monitoring explained only 53% of the variation in HbA_1c_ [30]. This suggests that HbA_1c_ may be determined by non-glycaemic factors. Glucose-independent factors such as red cell turnover and iron deficiency are known to affect HbA_1c_ concentration. Cohen *et al*. have demonstrated that variation in mean erythrocyte age, even in haematologically normal individuals, can significantly affect the time available for glycation and therefore the HbA_1c_ concentration [31]. Iron-deficiency anaemia has been associated with higher HbA_1c_ levels, with reductions after iron replacement therapy [32]. A recent meta-analysis of 23 genome-wide association studies identified ten genomic loci associated with HbA_1c_. Of these, seven were unrelated to glycaemic pathways, and influenced HbA_1c_ via their effects on iron status and red cell indices [33]. These studies highlight the contributions of non-glycaemic factors to HbA_1c_ levels and may potentially explain the observed ethnic differences in HbA_1c_.

The strengths of this study include a large sample size comprising individuals without a known diagnosis of diabetes mellitus, allowing the results to be extrapolated to the general population. By examining Chinese, Malay and Indian ethnic groups, this study also provides important information for other countries in Asia where these ethnic groups predominate. This is especially relevant as Asia is expected to witness a doubling in the numbers of people with diabetes by 2030 [21]. The ranges of fasting plasma glucose and HbA_1c_ values obtained were wide, which allowed exploration of the relationship between ethnicity, fasting plasma glucose and HbA_1c_ across the normal, impaired glucose and diabetic spectrum of values.

This study also has several limitations. Firstly, we did not capture the full range of glucose variability. Only a single point measurement of fasting plasma glucose was used, whereas the HbA_1c_ represents the average level of glycaemia over several months. Failure to capture the intra-individual variability of fasting plasma glucose may have led us to underestimate the proportion of variance in HbA_1c_ explained by fasting plasma glucose. Nevertheless, only one or two glucose measurements taken over a relatively short time are used to diagnose diabetes and, as such, we believe that our findings remain clinically important. We also failed to fully capture the non-fasting blood glucose. This has an impact on our ability to fully appreciate the relationship between blood glucose and HbA_1c_ between ethnic groups. However, as most guidelines now recommend fasting plasma glucose as the diagnostic test of choice for the diagnosis of diabetes, our findings remain relevant to the diagnosis of diabetes. Thirdly, although our findings suggest that the use of HbA_1c_ for diagnosis will result in reclassification of different numbers of individuals in different ethnic groups, it remains unclear if this reclassification does or does not result in the appropriate identification of persons at higher risk of diabetes-associated complications. As a consequence, we cannot interpret these data as suggesting that ethnic-specific cut-offs are required for the diagnosis of diabetes. Long-term prospective data will be required to draw such a conclusion. Finally, other non-glycaemic factors known to influence HbA_1c_ were not studied. Our study, however, captures the variation that may be observed in the usual clinical setting where such detailed profiling will be rarely undertaken. Given that most clinicians will not have access to information on these parameters for individual patients presenting to them for glycaemic testing, knowledge and awareness that ethnicity can introduce this magnitude of difference in HbA_1c_ levels may be important in decision making.

In summary, we have shown that Malays and Indians have a higher HbA_1c_ than Chinese at the same fasting plasma glucose level. In addition, as fasting plasma glucose increases, HbA_1c_ increases more rapidly in Malays and Indians than in Chinese. The use of HbA_1c_, in place of fasting plasma glucose, will reclassify different proportions of the population in different ethnic groups. Further studies are required to understand the glycaemic and non-glycaemic effects of ethnicity on HbA_1c_ and diabetes-associated complications, prior to determining whether ethnic specific cut-offs for HbA_1c_ are appropriate for the diagnosis of diabetes mellitus.

## Supporting Information

**Table S1.** Additional characteristics of participants in the study.
