# Supplementary material for: Ethnicity modifies the relation between fasting plasma glucose and HbA1c in Indians, Malays and Chinese
Source: Diabet Med. 2012 Jul;29(7):911–7. doi: 10.1111/j.1464-5491.2012.03599.x (PMC3504343; doi:10.1111/j.1464-5491.2012.03599.x)
Supplement: Supplementary file 1 [file dme0029-0911-SD1.docx]

Supplementary Table 1 – Additional characteristics of participants in the study

| **Parameter** | **All** | **Chinese**  **(N=2697, 69.2%)** | **Malay**  **(N=633, 16.3%)** | **Indian**  **(N=565, 14.5%)** | ***P*** |
| --- | --- | --- | --- | --- | --- |
| **Smoking (Yes)** | 471 (12.1) | 288 (10.7) | 115 (18.2) | 67 (11.9) | <0.001 |
| **Alcohol (Yes)** | 997 (25.6) | 855 (31.7) | 13 (2.1) | 129 (22.8) | <0.001 |
| **Family history DM** |  |  |  |  | <0.001 |
| **0** | 2553 (65.5) | 1864 (69.1) | 418 (66.0) | 269 (47.6) |  |
| **1** | 1034 (26.5) | 675 (25.0) | 164 (25.9) | 195 (34.5) |  |
| **≥ 2** | 310 (8.0) | 158 (5.9) | 51 (8.1) | 101 (17.9) |  |
| **HT (history)** | 784 (18.6) | 494 (18.3) | 116 (18.3) | 113 (20) | 0.32 |
| **HT ( History + measurement )** | 1473 (37.8) | 975 (36.2) | 267 (42.2) | 229 (40.5) | 0.006 |

Abbreviations: DM, diabetes mellitus; HT, hypertension
